# Supplementary material for: COVID-19 in Pregnancy: Knowledge about the Vaccine and the Effect of the Virus. Reliability and Results of the MAMA-19 Questionnaire
Source: Int J Environ Res Public Health. 2022 Nov 12;19(22):14886. doi: 10.3390/ijerph192214886 (PMC9690879; doi:10.3390/ijerph192214886)
Supplement: Supplementary file 1 [file ijerph-19-14886-s001.zip › File S1.pdf]

English translation of thE MAMA-19 questionnaire on the basis of the results obtained.

MAMA19

QUESTIONNAIRE ON COVID-19 VACCINATION DURING PREGNANCY AND PUERPERIUM: EFFECTIVENESS OF COMMUNICATION BY INSTITUTIONS AND THE CLINICAL WORLD

Dear Madam,

We would like you to participate to this anonymous survey concerning the COVID-19 vaccination during pregnancy and post-partum. The purpose is to investigate the effectiveness of risk communication in health care in particular crisis situations. We ask you to answer freely and truthfully, you will not be judged in any way. The results from the completed questionnaires will be used for the elaboration of a Master's Degree thesis.

Thank you!Grazie

Section S1. General Information

S1.1.Age ....

S1.2. Civil status

□ Single

□ Married or with a partner

□ Divorced or Separated

□ Widowen

S1.3. Educational level

□ None
□ Primary school
□ secondary school
□ high school
□ Graduated

S1.4. Which region of Italy do you live in? ………………………………..

S1.5. Nationality

□ Italian
□ Other (specify) ……………………………………

S1.6. Are you currently employed?

□ Yes
□ No

S1.7. If you have a partner or married. Is your partner working?

- Yes
- No

S1.8. How many conceptions have you had in your life?

□ **1**
□ 2
□ 3
□ 4 or more

S1.9. Gestational age in weeks (number): …………

S1.10. Type of birth:

□ Vaginal birth
□ Cesarean birth

S1.11. Number of sons before this delivery:

□ 1
□ 2
□ 3 or more

S1.12. During this pregnancy how many obstetrical visits did you have?

□ Less than 4
□ Form 4 to 9 (about one per month)
□ More than 9 (it happened more than 9 per month)

S1.13. Did you receive a flu vaccination during pregnancy?
□ Yes
□ No

S1.13A. If no, what is the reason for non-vaccination?
□ I was not recommended to do it
□ I afraid of side effects on myself and/or the child
□ I think that it isn't necessary for me
□ I could not find a place to do it
□ Other reason (specify) …………………………………………………………

S1.14. Before you knew you were pregnant, were you concerned about COVID19 infection for yourself before pregnancy?

- Yes
- No

S1.15. Before you knew you were pregnant, were you concerned about COVID19 infection for people you care about (parents, partner, relatives, other children, friends, etc.) before your pregnancy?

- Yes
- No

Section S2. KNOWLEDGE ABOUT EFFECT of COVID-19 VACCINATION DURING PREGNANCY

Please choose a value from 1 to 5 (1=strongly disagree; 2=disagree; 3=neutral; 4=agree; 5=strongly agree)

S2.1 Pregnant women who get COVID-19 vaccination risk to have:

S2.1A Neonates malformations

1 2 3 4 5

S2.1B Premature delivery

1 2 3 4 5

S2.1C Intra Uterin Growth Restriction (IUGR) 1 2 3 4 5

S2.2 Pregnant women not vaccinated against COVID-19 have the same complications, in case of a positive test result, as vaccinated pregnant women

□ Don't know
□ true
□ false

S2.3 COVID-19 vaccine reduces mortality

□ Don't know
□ true
□ false

S2.4 COVID-19 vaccine reduces risk of hospitalization

- Don't know
- true
- false

S2.5 COVID-19 vaccine reduces short-term infectiousness

- Don't know
- true
- false

S2.6 COVID-19 vaccine reduces mortality during pregnancy

- Don't know
- true
- false

SECTION S3. KNOWLEDGE ABOUT EFFECT OF COVID-19 INFECTIONS DURING THE PREGNANCY

S3.0 Pregnant women have a higher risk of complications from COVID-19 infection than women who are not pregnant

- Don't know
- Same risk
- True
- Partial true, only in certain cases

S3.1 COVID-19 infection can be highly contagious

□ No
□ Yes
□ Don't Know

S3.2 Being infected with COVID-19 may require hospitalisation

- No
- Yes, in certain cases
- Yes, always
- I don't know

S3.3 Possible post COVID-19 consequences in the unvaccinated: (possible more answers)

- Excessive tiredness and fatigue
- Headache
- Attention and memory disorders
- Abnormal hair loss
- Shortness of breath and breathing difficulties
- Cough
- Anosmia and ageusia
- Joint pain
- Abnormal sweating
- Chest pain
- Excessive fatigue after physical activity
- Nausea and vomit
- Memory loss

SECTION S4. SOURCES OF INFORMATION USED ON VACCINATION AND IMPACT OF COVID-19 DISEASE

S4.1 I received information on the effects of COVID-19 disease in pregnancy from: (possible more answers)

□ Health personnel (general practitioner, gynaecologist, obstetrics, etc.)
□ No health personnel (friends, parents, etc.)
□ My self (internet/TV/ etc.)
□ I didn't inform

S4.2 I have used institutional media to get information about COVID-19 vaccination in pregnancy. (Istitutional media are web site, documents, papers or televison programs sponsorized fromMinister of health, Local Health Unit, etc.).

□ Yes
□ No

S4.3 If yes, institutional media used to obtain information on COVID-19 vaccination in pregnancy: (possible more answers**)**

□ Internet
□ TV
□ Newspapers
□ Journals
□ Radio
□ Green number / call center dedicated
□ Other (specify) …………………………………………………………………….

S4.4 A I have approached professionals for information about COVID-19 vaccination in pregnancy (general practitioner, gynaecologist, obstetrics, etc)

□ Yes
□ No

S4.4B I have approached professionals for information about COVID-19 disease in pregnancy (general practitioner, gynaecologist, obstetrics, etc)

□ Yes
□ No

S4.5 Which professionals did you turn to: (possible more answers**)**

- General practitioner
- Gynecologist
- Midwife
- Nurse
- Pharmacist
- ASLd
- Childbirth preparation course

- Vaccination centre

S4.6 I'm sufficiently satisfied with communication with professionals: (possible more answers)

- General practitioner
- Gynecologist
- Midwife
- Nurse
- Pharmacist
- ASL
- Childbirth preparation course
- Vaccination centre

S4.7 If you have consulted more than one professional, have you received consistent information?

- Yes
- no

SECTION 5. PERSONAL POSITION ABOUT COVID-19 VACCINATION

S5.1 Have you been vaccinated (two or more doses) for COVID-19??

□ yes
□ No

S5.2 If yes, when did you have your first dose?

- □ Before pregnancy
- □ I trimester
- □ II e III trimester

S5.3 If no, wants to vac-cinate, what is the reason for not vaccinating?

- □ Poor knowledge
- □ Fear
- □ Previous COVID-19
- □ Healthcare providers does not
- □ My parents/ relatives suggest to me no vaccine
- □ For my patologies ….
- □ I'm wating
- □ Other specify ………………………………………………………

S5.4 If you are waiting, what is the reason for the delay in vaccination?

- □ After the end of breastfeeding
- □ I am not convinced about the effectiveness of the vaccine
- □ I did not know about the possibility of the vaccine during the pregnancy
- □ Waiting term of pregnancy
- □ Previous COVID-19

Thanks for your collaboration
