# Supplementary material for: COVID-19 in Pregnancy: Knowledge about the Vaccine and the Effect of the Virus. Reliability and Results of the MAMA-19 Questionnaire
Source: Int J Environ Res Public Health. 2022 Nov 12;19(22):14886. doi: 10.3390/ijerph192214886 (PMC9690879; doi:10.3390/ijerph192214886)
Supplement: Supplementary file 1 [file ijerph-19-14886-s001.zip › File S2.pdf]

Source: Mappa I, Luviso M FA, Disefano MA, et al. Women perception of SARS-CoV-2 vaccination during pregnancy and subsequent maternal anxiety: a prospective observational study. J Matern Fetal Neonatal Med 2021;1-4. https://doi.org/10.1080/14767058.2021.1910672 Supplementary material page 3.

STAI QUESTIONNARIE

PART A

1) Did you get the flu vaccination during pregnancy?

- Yes
- No
- I'm waiting to run it

2) In the answer is no to the previous question, what is the reason for not vaccinating?

- I was not advised to do so

- I was afraid of the side effects on me and the baby

- I do not consider it necessary

- I couldn't find it

3) Did you have information on the effects of Covid-19 (SARS-COV 2) in pregnancy?

- No

- Yes from my gynaecologist, general practioner

- I read on internet/ Journal, Television

4) Did you have any information on the effects of Covid 19 vaccine in pregnancy?

- No

- Yes from my gynaecologist, general practioner

- I read on internet/ Journal, Television

5) In a range of 1-5, how worried are you that the Covid 19 vaccine could cause malformation on your baby?

6) In a range of 1-5, how worried are you that the Covid 19 vaccine could cause premature birth?

7) In a range of 1-5, how worried are you that the Covid 19 vaccine could cause growth restriction?

8) Covid 19 infection is highly contagious?

- Surely not

- I do not think so

- I do not know

- I think so

- Surely

9) Covid 19 infection can sometimes be severe enough to require hospitalization?

- Surely not
- I do not think so
- I do not know
- I think so
- Surely

10) Young children are at higher risk of having to be hospitalized due to Covid 19 infection than adults

- Surely not
- I do not think so
- I do not know
- I think so
- Surely

11) Pregnant women run the same risk of complications from Covid 19 infection as women who are not pregnant?

- Surely not
- I do not think so
- I do not know
- I think so
- Surely

12) The Covid 19 vaccine is safe?

- Surely not
- I do not think so
- I do not know
- I think so
- Surely

13) The Covid 19 vaccine is effective?

- Surely not
- I do not think so
- I do not know
- I think so
- Surely

14) The Covid 19 vaccine is safe in pregnancy?

- Surely not
- I do not think so
- I do not know
- I think so
- Surely

15) You would like to have the anti-covid 19 vaccination during pregnancy?

- Surely not
- I do not think so
- I do not know
- I think so
- Surely

16) What importance do you give to the start of anti-Covid 19 vaccinations?

- turning point in the pandemic
- useless
- propaganda
- I have no idea
