# Supplementary material for: COVID-19 in Pregnancy: Knowledge about the Vaccine and the Effect of the Virus. Reliability and Results of the MAMA-19 Questionnaire
Source: Int J Environ Res Public Health. 2022 Nov 12;19(22):14886. doi: 10.3390/ijerph192214886 (PMC9690879; doi:10.3390/ijerph192214886)
Supplement: Supplementary file 1 [file ijerph-19-14886-s001.zip › File S3.pdf]

The vaccination Attitudes Examination Scale (VAX-I)

Versione Italiana

Istruzioni. Queste domande sono progettate per aiutarci a comprendere meglio le convinzioni delle persone sulle vaccinazioni. Le chiedo di contrassegnare le risposte che riflettono più accuratamente i suoi sentimenti o le sue convinzioni. Non ci sono risposte giuste o sbagliate.

1. Mi sento al sicuro dopo essere stata/o vaccinata/o. (-)	Fortemente in Disaccordo 1	2	3	4	5	Fortemente D'accordo 6
2. Posso contare sui vaccini per fermare le malattie infettive gravi. (-)	Fortemente in Disaccordo 1	2	3	4	5	Fortemente D'accordo 6
3. Mi sento protetto/a dopo essermi vaccinato/a. (-)	Fortemente in Disaccordo 1	2	3	4	5	Fortemente D'accordo 6
4. Sebbene la maggior parte dei vaccini sembri sicura, potrebbero esserci dei problemi non ancora scoperti.	Fortemente in Disaccordo 1	2	3	4	5	Fortemente D'accordo 6
5. I vaccini possono causare problemi imprevedibili nei bambini.	Fortemente in Disaccordo 1	2	3	4	5	Fortemente D'accordo 6
6. Mi preoccupano gli effetti sconosciuti che i vaccini potrebbero avere in futuro.	Fortemente in Disaccordo 1	2	3	4	5	Fortemente D'accordo 6
7. I vaccini fanno guadagnare molti soldi alle aziende farmaceutiche, ma in realtà non sono utili alle persone.	Fortemente in Disaccordo 1	2	3	4	5	Fortemente D'accordo 6
8. Le autorità promuovono la vaccinazione a scopo di lucro e non per la salute delle persone.	Fortemente in Disaccordo 1	2	3	4	5	Fortemente D'accordo 6
9. I programmi di vaccinazione sono una grande truffa.	Fortemente in Disaccordo 1	2	3	4	5	Fortemente D'accordo 6
10. L'immunità naturale dura più a lungo di una vaccinazione.	Fortemente in Disaccordo 1	2	3	4	5	Fortemente D'accordo 6
11. L'esposizione naturale a virus e germi offre la protezione più sicura.	Fortemente in Disaccordo 1	2	3	4	5	Fortemente D'accordo 6
12. Per il sistema immunitario è più sicuro essere esposti in modo naturale alle malattie piuttosto che tramite la vaccinazione.	Fortemente in Disaccordo 1	2	3	4	5	Fortemente D'accordo 6

Calcolo dei punteggi. I punteggi della scala e delle sottoscale si ottengono dalle medie dei punteggi degli item (Item 1, 2, 3 = F1: sfiducia nei benefici dei vaccini; Item 4, 5, 6 = F2: preoccupazione per futuri effetti imprevisti; Item 7, 8, 9 = F3: preoccupazione per i profitti commerciali; Item 10, 11, 12 = F4: preferenza per l'immunità naturale). (-) Codifica inversa

VAX questionnaire (original version)

Source: Martin, L.R.; Petrie, K.J. Understanding the dimensions of anti-vaccination attitudes: The vaccination attitudes examination (VAX) Scale. *Ann. Behav. Med.* **2017**, *51*, 652–660.

Study 1 VAX scale items factor loading matrix (EFA)

Items	Mistrust of vaccine benefit
I feel safe after being vaccinated (–)	**.799**
I can rely on vaccines to stop serious infectious diseases (–)	**.711**
I feel protected after getting vaccinated (–)	**.761**
Although most vaccines appear to be safe, there may be problems that we have not yet discovered.	.142
Vaccines can cause unforeseen problems in children.	.227
I worry about the unknown effects of vaccines in the future.	.139
Vaccines make a lot of money for pharmaceutical companies, but do not do much for regular people.	.202
Authorities promote vaccination for financial gain, not for people's health.	.251
Vaccination programs are a big con.	.286
Natural immunity lasts longer than a vaccination.	.112
Natural exposure to viruses and germs gives the safest protection.	.081
Being exposed to diseases naturally is safer for the immune system than being exposed through vaccination.	.190
